# Changes in gene expression in chronic allergy mouse model exposed to natural environmental PM2.5-rich ambient air pollution

**DOI:** 10.1038/s41598-018-24831-z

**Published:** 2018-04-20

**Authors:** Yuhui Ouyang, Zhaojun Xu, Erzhong Fan, Ying Li, Kunio Miyake, Xianyan Xu, Luo Zhang

**Affiliations:** 1 Department of Otolaryngology Head and Neck Surgery and department of Allergy, Beijing TongRen Hospital, Affiliated to the Capital University of Medical Science, Beijing, 100730 China; 2 Beijing Key Laboratory of Nasal Diseases, Beijing Institute of Otolaryngology, Beijing, 100005 China; 3 Department of Environmental Medicine, Quanzhou Medical College, Quanzhou, Fujian, 362011 China; 4 Department of Biochemistry, Interdisciplinary Graduate School of Medicine and Engineering, University of Yamanashi, Yamanashi, 409-3898 Japan; 5 Department of Health Sciences, Graduate School of Interdisciplinary Research, University of Yamanashi, Yamanashi, 409-3898 Japan

## Abstract

Particulate matter (PM) air pollution has been associated with an increase in the incidence of chronic allergic diseases; however, the mechanisms underlying the effect of exposure to natural ambient air pollution in chronic allergic diseases have not been fully elucidated. In the present study, we aimed to investigate the cellular responses induced by exposure to natural ambient air pollution, employing a mouse model of chronic allergy. The results indicated that exposure to ambient air pollution significantly increased the number of eosinophils in the nasal mucosa. The modulation of gene expression profile identified a set of regulated genes, and the Triggering Receptor Expressed on Myeloid cells1(TREM1) signaling canonical pathway was increased after exposure to ambient air pollution. *In vitro*, PM2.5 increased Nucleotide-binding oligomerization domain-containing protein 1 (Nod1) and nuclear factor (NF)-κB signaling pathway activation in A549 and HEK293 cell cultures. These results suggest a novel mechanism by which, PM2.5 in ambient air pollution may stimulate the innate immune system through the PM2.5-Nod1-NF-κB axis in chronic allergic disease.

## Introduction

Environmental pollution is a major problem in China, especially Beijing; with increased number of vehicles, heating in the winter season, and industrial emissions being major contributors to airborne particulate matter (PM). PM2.5, with an aerodynamic diameter ≤2.5 µm, is a complex mixture containing toxic and/or carcinogenic chemicals, including acids, inorganic ions, metals, and poly aromatic hydrocarbons (PAHs)^1^ which can penetrate the alveoli of the lungs. Several studies have indicated that PM2.5 exposure is associated with overall mortality and morbidity and increased risk of lung cancer^2–4^. Epidemiologic evidence has suggested that PM2.5 may lead to cardiovascular disease, respiratory disease, chronic obstructive pulmonary disease (COPD), and asthma or rhinitis, as well exacerbate the allergic response^5,6^. A recent study has suggested that particulates act as adjuvants and promote long-lasting allergic inflammation in the airways^7^. Studies investigating the potential mechanisms underlying the adverse effects of exposure to PM2.5 have suggested that these include inflammation, oxidative stress, and apoptosis response^8^. Although some studies have investigated the effect of natural exposure to PM2.5 on the health of traffic policeman or employees at trucking terminals^9,10^, the precise mechanism/s linking the effects of PM2.5 pollution in real-life settings on human health have not yet been elucidated.

Modulation of gene expression has been shown to play a major role in the activation of toxic pathways, and in this regard gene signatures have the potential to act as biomarkers of PM2.5 exposure. Indeed, several studies have reported changes in gene expression following PM2.5 exposure. Chu and colleagues^10^ have recently demonstrated that several genes and gene networks; many of which have been implicated in ischemic heart disease, chronic obstructive pulmonary disease (COPD), lung cancer, and other pollution-related illnesses; were differentially significantly activated in response to PM2.5, elemental carbon (EC), and organic carbon (OC) in the trucking industry employees. Similarly, exposure to Milan PM2.5 has been shown to alter gene expression and the production of reactive oxygen species (ROS) in human alveolar epithelia cells (A549), and suggested that DNA damage was likely to be related to changes in the cytochrome P450 (CYP) enzyme activity^11^. Analysis of microRNA and mRNA changes in A549 cells exposed to water and organic soluble extract from PM2.5 has further indicated that the predicted target genes were involved in the regulation of cell cycle^12^. A more recent study has demonstrated sex-specific candidate gene expression in response to PM10 exposure in humans^13^.

In the present study, we have investigated the cellular responses induced by exposure to ambient PM2.5-enriched air pollution in a mouse model of chronic allergy. We have also performed genome-wide gene expression microarray analysis using whole blood RNA and analysis of gene expression networks by Ingenuity Pathway Analysis (IPA). Our data showed that “TREM1 (Triggering Receptor Expressed on Myeloid cells1) signaling” is a high-rank canonical pathway. PM2.5 exposure induced the intracellular pattern recognition receptor Nod1 activity and stimulated an immune reaction by the activation of NF-κB signaling pathway.

## Materials and Methods

### Animals

Female BALB/c mice (8–10 weeks) were obtained from the Faculty of Laboratory Animals of Capital Medical University and maintained under pathogen-free, clean air conditions; with water and standard laboratory diet supplied *ad libitum*. The mice were divided into four groups (n = 15). Two control groups consisted of mice that received clean air with ovalbumin (OVA) challenge (termed CA) or without OVA challenge (termed CC). The other two groups were exposed to ambient air pollution; during the period between 1^st^ September to 20^th^ October; with OVA challenge (termed AP) or without OVA challenge (termed CP).

The study was conducted in full accordance with Declaration of Helsinki and was approved by the Animal Ethics Committees at the Beijing Institute of Otolaryngology and the University of Yamanashi.

### PM2.5 and pollen sampling

PM2.5 were collected using a sampler (TH-16A, Wuhan Tianhong Instruments Co., Ltd. Wuhan, China) located at the roof top of Beijing Tongren Hospital Building (16°41′*N*, 39°90′*E*, 10 m above the ground) in the Chongwenmen district, and situated 150 m from the main road and 500 m from the train station. Samples of PM2.5 were collected during the period between 1^st^ September to 20^th^ October, when the animals were exposed to the ambient air pollution and the PM2.5 concentrations in the air ranged from 8.57 to 269.62 μg/m^3^ (average, 89.38 μg/m^3^). Briefly, ambient air was drawn into the sampler at an average flow rate of 1.13m^3^/min for 24 h (9:00 AM 9:00 AM the next day) and PM2.5 samples were collected on 37 mm Teflon filters. Post sampling, the filters were sterilized, weighed, cut into small pieces, and immersed in three volumes of deionized water in conical bottom Falcon tubes (Becton Dickenson, Franklin Lakes, NJ, USA). The samples were then sonicated for 4 × 30 min periods in a sonication bath (TA4905, Tamagawa Seiki, Nagano, Japan), and the extracted PM2.5 particles were dried and weighed, prior to being solubilized in sterilized water at a final concentration of 20 μg/μL. The samples were stored at −80 °C until further use.

Pollen samples of Oak (Quercusmongolica), a tree species that produces catkins each year during the spring months (usually around April) in Beijing, China, were collected as described previously^14^. Anthers from oak trees were collected from the Botanical Garden of the Chinese Academy of Sciences and dried at 27 °C. The pollens were released by gently crushing the dried anthers and sieving through 50-μm mesh, prior to storage at −20 °C. Dried pollen were suspended in 1 mL deionized water, and agitated on a test tube shaker for 48 h at 4 °C. The pollen solutions were centrifuged at 1000 g for 15 min at 4 °C and the supernatants collected for estimation of the protein concentration, by bicinchoninic acid (BCA) method (Thermo Fisher Scientific, Carlsbad, CA, USA). The samples were stored at −80 °C until further use.

### Chronic allergy mouse model and PM2.5-rich ambient air pollution exposure

**Figure 1:**
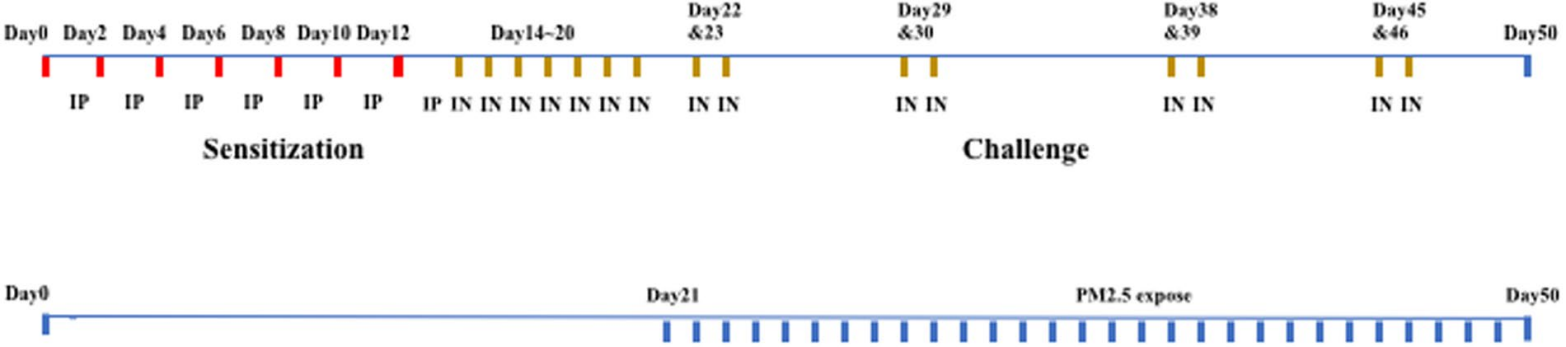
Study protocols. Sensitization, challenge, and air pollution exposure protocols for chronic allergy experiment.

A chronic allergy mouse model was established as described previously^15,16^. Briefly, mice allocated to CA and AP groups were sensitized by intraperitoneal injection of 50 µg ovalbumin (OVA, Sigma Aldrich, St. Louis, MO, USA) in the presence of 5 mg aluminum hydroxide solubilized in 1 mL sterile saline on days 0, 2, 4, 6, 8, 10, and 12. Starting on day 14, the mice were challenged with 5% intranasal OVA (10 µL/side) once a day for 7 days, and then twice weekly for 4 weeks from day 22 (Fig. 1). Control mice (groups CC and CP) were challenged with intranasal phosphate-buffered saline (PBS) in a similar manner.

A model to investigate the effect of exposure to ambient concentrations of PM2.5-rich in mice with chronic allergy was established by exposing the CP and AP mice to air pollutants in the natural environment in Beijing from day 22, in combination with intranasal challenge with 5% OVA. The groups of CP and AP mice were exposed to ambient outdoor air pollution near major roads for 8 h a day for 4 weeks during the period between 1^st^ September to20^th^ October, when the seasonal concentration of PM2.5 in Beijing is high. For the purpose of this study, exposure was performed at the site where the PM2.5 sampler was situated, as described above.

### Histology

24 h after the final nasal challenge, the animals were executed using CO_2_. The nasal tissues were excised and fixed with 10% (v/v) formaldehyde prior to embedding in paraffin. The coronal nasal sections were visualized by hematoxylin and eosin (H&E) staining for inflammatory cell accumulation, and by periodic acid-Schiff (PAS)/hematoxylin staining for mucus secretion^15^. The number of infiltrating eosinophils in the nasal mucosa in the posterior portion of nasal septum was determined microscopically in a blinded manner and expressed as the number per high-power field (400×). Two specimens of Hansel-stained coronal sections from each mouse were selected for assessment and the mean score was calculated for 6 animals.

### Microarray and bioinformatic data analysis

Total RNA was extracted from blood using RNeasy Protect Animal Blood Kit according to the manufacturer’s directions (Qiagen, Valencia, CA, USA). The isolated total RNA was subjected to DNA microarray analysis using the Mouse Gene 1.0 ST Array (Affymetrix) according to the manufacturer’s instructions. The microarray data were normalized using the Robust Multiarray Average (RMA) algorithm. The raw data were deposited in the Gene Expression Omnibus (GEO) database; accession number GSE46405; and the lists of genes modulated by PM2.5 exposure were investigated by IPA (Ingenuity QIAGEN Redwood City, CA, USA) to identify the gene networks based on the biological functions and/or diseases.

### Cell culture and treatment

Human alveolar epithelial cells (A549) and HEK293-Nod1-GFP cells (kind gift from Dr. Naohiro Inohara, University of Michigan Medical School, Ann Arbor, Michigan, USA) were cultured in 6-well culture plates (Thermo Fisher Scientific), at a density of 1.5 × 10^5^ cells in Dulbecco’s Modified Eagle Medium (DMEM) (Gibco, Grand Island, NY, USA) supplemented with 10% Fetal Bovine Serum (FBS), 1% penicillin/streptomycin (10,000 U/mL) at 37 °C in an atmosphere of 5% CO_2_ and treated with 350 μg/mL PM2.5 for 24 h. For the purpose of this experiment a total of three cultures were used to test the effect of each treatment. The HEK293-Nod1-GFP cells, a cell line which can constitutively express Nod1-Flag and NF-κB-dependent green fluorescent protein (GFP) reporter, was used for assessment of Nod1 activity as demonstrated by Hasegawa and colleagues^17^. The Nod1 triggered NF-κB-dependent transcription activity was determined by measurement of GFP intensity using a fluorescence plate reader (SpectraMax GeminiEM, Molecular Devices, Sunnyvale, CA, USA), at excitation 485 nm and emission 530 nm.

### Western blot

A549 cells were cultured in 6-well plates at a density of 1.5 × 10^5^ cells, as above, and after 1 day of culture, the cells were treated with PM2.5 or pollen for 24 h. For the purpose of this experiment a total of three cultures were used to test the effect of each treatment. The cells were lysed in a buffer containing 1% Nonidet P-40, 20 mM Tris-HCl (pH 7.4), 150 mM NaCl, 1 mM PMSF, 1% aprotinin, and 5 mM EDTA, and the protein concentrations of the lysates were detected by Pierce BCA Protein Assay (Thermo Fisher Scientific). The proteins were subjected to SDS-PAGE, and the separated protein bands were transferred to Immobilon-P membrane (Merck Millipore, Billerica, MA, USA), for immunoblotting using the indicated antibodies. The immunoreactive bands were detected using the electrochemiluminescence (ECL) system (Amersham, UK) and visualized using a lumino image analyzer (RAS-1000; Fuji Film, Tokyo, Japan).

### Quantitative Real-time Polymerase Chain Reaction (qRT-PCR)

Total RNA from the cultured cells was extracted using RNeasy Plus Mini Kit according to manufacturer’s directions (Qiagen, Valencia, CA, USA). First-strand cDNA was synthesized using PrimeScript reverse transcriptase (Takara Bio, Shiga, Japan) and oligo (dT) 12–18 primers (Invitrogen, Carlsbad, CA, USA). qRT-PCR analysis was performed using Platinum SYBR Green qPCR SuperMix-UDG with ROX (Invitrogen) and the ABI PRISM 7000 Sequence Detection System (Applied Biosystems, Foster city, CA, USA). The specificity of the detected signals was confirmed via dissociation protocol. All samples were run in duplicate in each experiment and the values were normalized against the levels of glyceraldehyde-3-phosphate dehydrogenase (*GAPDH*) mRNA. The primers used were as follows: mouse *GAPDH* (sense, 5′-TGCAGTGGCAAAGTGGAGATT-3′; antisense, 5′-TGCCGTTGAATTTGCCGT-3′); mouse *Nod1* (sense, 5′-CCAAAGCCCGACAGAAACTC-3′; antisense, 5′-CAGCATCCACAGGAATGTGG-3′).

### Statistical analysis

Microarray and bioinformatic data was analyzed using the Mouse Gene 1.0 ST Array and Gene Expression Omnibus (GEO) database. Data from each experiment were presented as mean values ± SEM and differences between groups were analyzed using the two-tailed Student’s t-test. Values of p < 0.05 were considered significant.

## Results

### Effect of natural exposure to PM2.5-rich ambient air pollution in the nasal mucosa of chronic allergy mouse model

**Figure 2:**
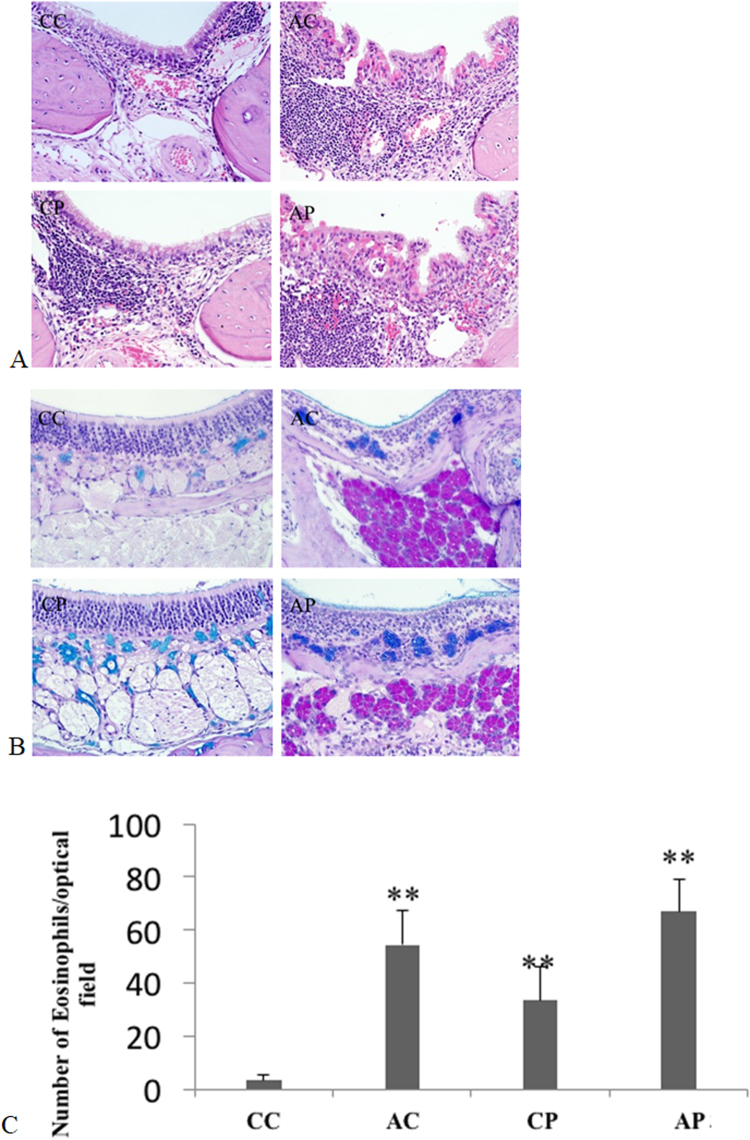
H&E- (**A**) and PAS-stained (**B**) nasal sections from chronically challenged mice. The number of infiltrating eosinophils in the nasal mucosa in the posterior portion of nasal septum(**C**). (n = 15 in each group).

Assessment of the effect of exposure to ambient air pollution, indicated prominent infiltration of eosinophils into the nasal mucosa, and epithelial cell damage in CA, CP, and AP mice groups (Fig. 2A). The number of infiltrating eosinophils per HPF was significantly increased in CA (p < 0.01), CP (p < 0.01), and AP mice (p < 0.01) compared to the controls (Fig. 2C), and in AP mice (p < 0.01) compared to CA and CP mice. An increase in mucus was indicated by increased PAS staining in the nasal mucosa of CA, CP, and AP mice, compared to the control groups (Fig. 2B), suggesting that ambient air pollution exposure increased mucus secretion in chronic allergic mice model.

### Modulation of gene expression with exposure to ambient air pollution in chronic allergy

**Figure 3:**
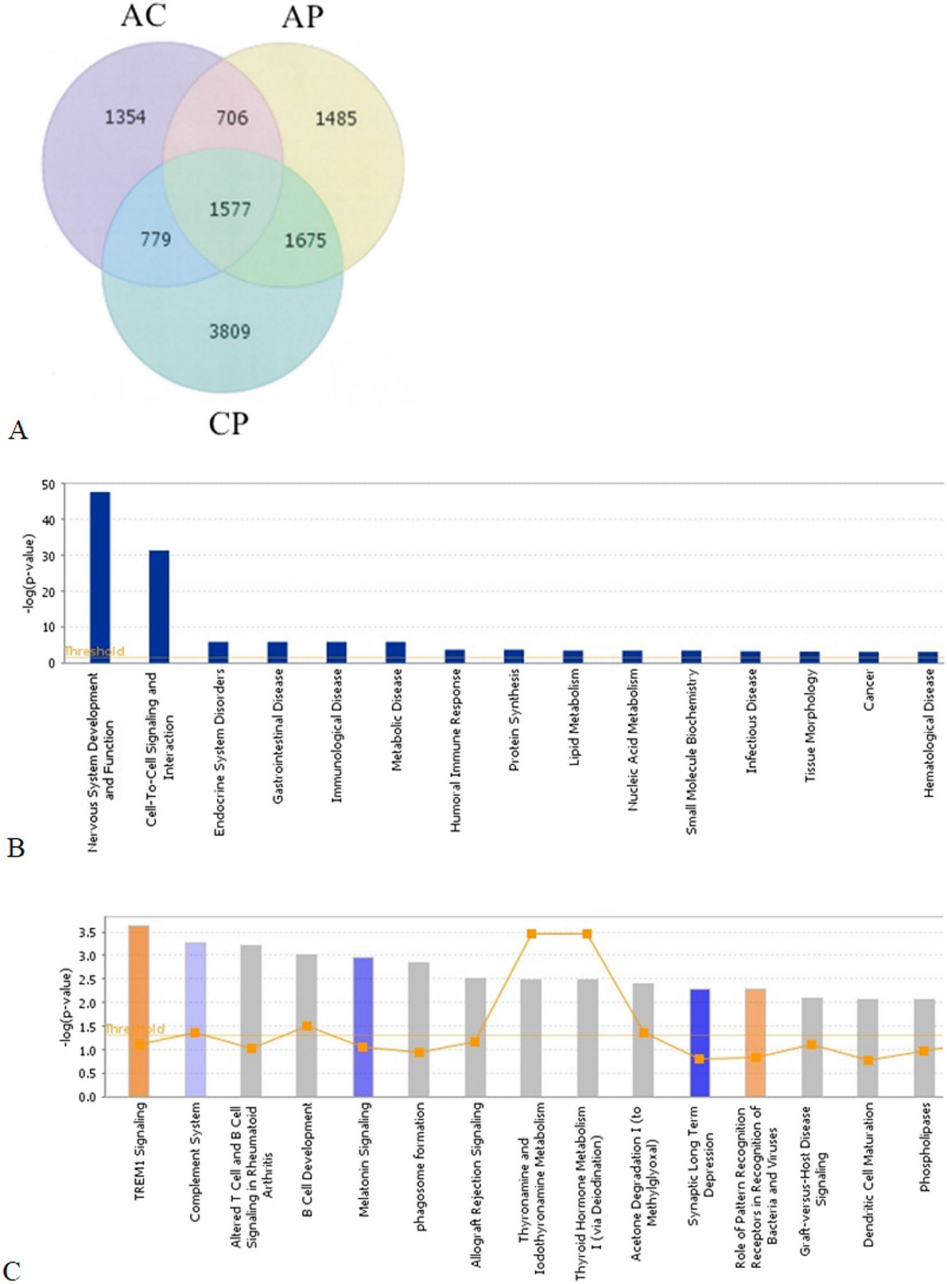
Venn diagram representing the numbers of common and air pollution exposure-specific regulated genes relative to clean air condition exposure (control). [*adjusted p*-*value* < *0*.*05*, *average expression level* (*AvgExp*) > *1*.*5 and log*_2_ *FC* (*fold change*) > *|0*.*7|*]. CC = clean air without OVA challenge, CA = clean air with OVA challenge, CP = ambient air pollution exposure without OVA challenge, AP = ambient air pollution exposure and OVA challenge (**A**). The main functional pathway (**B**) and main canonical pathway (**C**) of exposure to air pollution in chronic allergy mouse model, analysed by Ingenuity Pathway Analysis (IPA). The IPA was performed on DEGs to determine the air pollution exposure. The cut-off value of 1.5-fold was used for IPA. The left Y-axis in the figures denotes −log (*p*) values. The right Y-axis and curve in the figures denotes ratios of targets involved in one pathway.

Assessment of genome-wide gene expression profiles and molecular changes using whole blood RNA demonstrated that in comparison with the control mice, exposure of healthy mice to PM2.5-rich ambient air pollution (CP) significantly modulated gene expression of 7840 genes using 1.5-fold change as the cut-off value; of which 3918 genes were upregulated and 3922 downregulated. Similarly, exposure of the AP group (chronic allergy) modulated gene expression of 5443 genes; of which 2367 genes were upregulated and 3076 downregulated compared to the control group. Overall, 1675 genes were shared across the AP and CP groups, with 21.36% (1675/7840) and 30.77% (1675/5443) overlapping genes for the AP and CP groups, respectively; indicating that these overlapping genes had common molecular responses following exposure to ambient air pollution. Interestingly, 27.28% of the modulated genes in the AP group (1485/5443) presented only after exposure, thereby implicating them as a core set of genes in pathways related to chronic allergy following exposure to air pollution [Fig. 3A].

### Gene networks and function associated with exposure to ambient air pollution in chronic allergy

To understand the mechanism and function of differentially expressed genes (DEGs), IPA was performed to identify the gene networks based on the biological functions and/or diseases in the Ingenuity Pathways Knowledge Database. The global analysis results with IPA showed that the top five molecular and cellular functions of genes in the AP group were enriched targets in cell-to-cell signaling and interaction, protein synthesis, lipid metabolism; nucleic acid metabolism, and small molecule biochemistry [Fig. 3B]. A detailed analysis of the top five molecular and cellular functions of the up-regulated and down-regulated genes in AP are shown as Supplemental data 1 [Fig. S1]. The top five related diseases of target genes of AP included inflammatory response, immunological disease, gastrointestinal disease, organismal injury and abnormalities, and connective tissue disorders. An in-depth analysis of the top five target genes related diseases up- and down-regulated in AP is shown as Supplemental data 2 [Fig. S2].

The top fifteen canonical pathways of AP, according to the higher p*-*values are shown in Fig. 3C. In the main pathways of AP, those with high ratios included TREM1 signaling, complement system, altered T-cell and B-cell signaling in rheumatoid arthritis, and B-cell development. Interestingly, as the animals in the chronic allergy model were induced with OVA, the allograft rejection signaling and graft *vs*. host disease signaling was also shown to be the main canonical pathway in the AP group compared to the non-OVA treated CP group.

### The effect of PM2.5 exposure on expression and activation of Nod1

**Figure 4:**
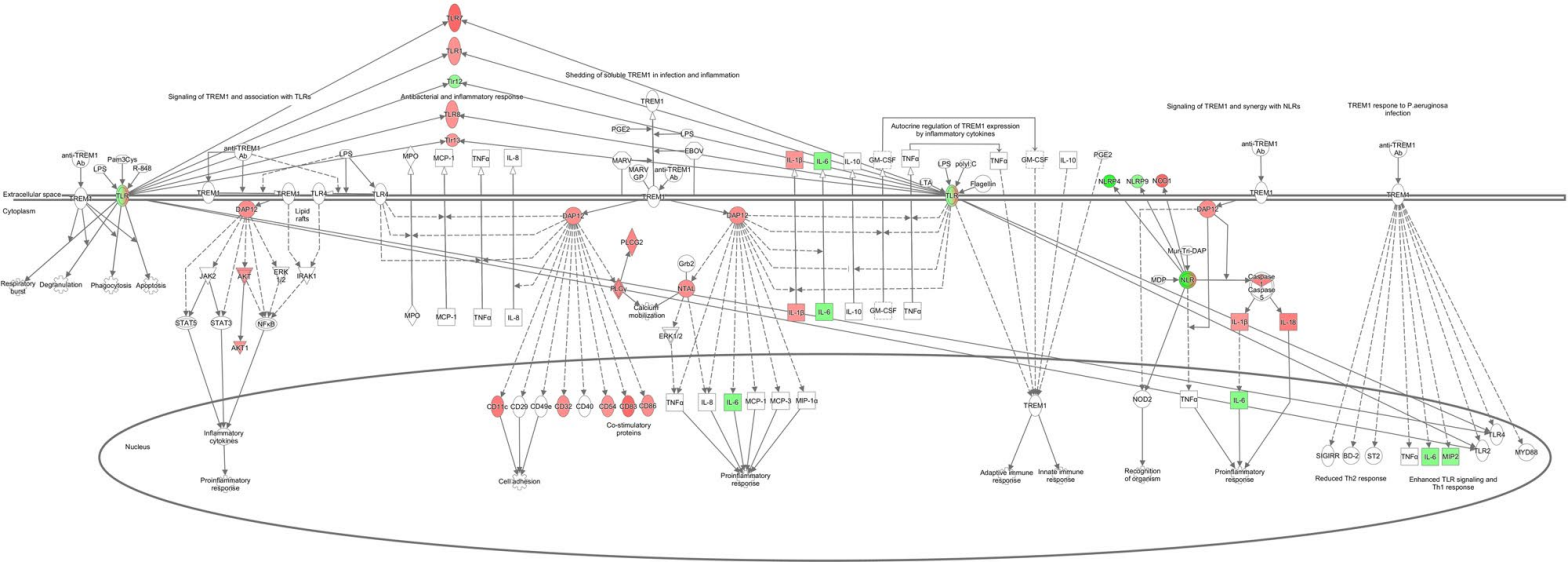
The signaling network of TREM1 and synergy with NLRs in chronic allergic mouse model exposed to PM2.5 assessed by IPA network analysis (QIAGEN Inc., https://www.qiagenbio-informatics.com/products/ingenuity-pathway-analysis). Green and red colors are associated to down-regulated and up-regulated genes, respectively. The intensity of the node color indicates the degree of up- or down-regulation. Direct and indirect interactions are represented by solid and dashed lines, respectively.

According to IPA, canonical pathway analysis of AP revealed that the “Role of Pattern Recognition Receptors in Recognition of Bacteria and Viruses” was presented as a higher rank of the canonical pathway in both CP and AP. The “TREM1 signaling” most significantly affected the canonical pathways in the AP group [Fig. 4]. Similarly, Nod1 was highly expressed as a result of exposure to air pollution, as indicated by microarray analysis, and involved in the signaling pathway of TREM1; via TREM-1 synergizing with Nod-like receptors (NLRs), as analyzed by IPA [Fig. 4].

**Figure 5:**
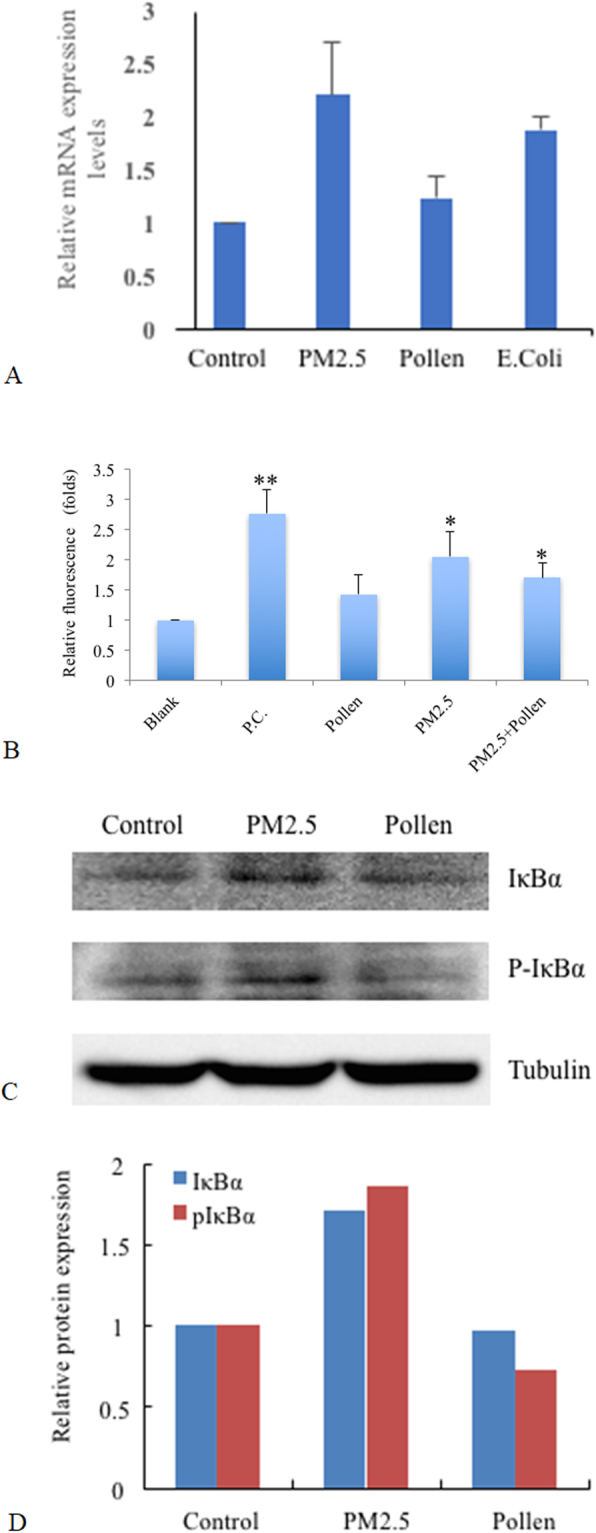
PM2.5 induces Nod1 expression and activates NF-κB signaling pathway. (**A**) The expression of Nod1 mRNA in A549 cells treated with PM2.5, pollen and *E*. *coli*, as assessed by qRT-PCR. (**B**) PM2.5 stimulated Nod1 activity in HEK293 cells constitutively expressing Nod1-Flag and NF-κB-dependent GFP reporter, as measured by fluorescence density. (**C**,**D)** Histograms and representative expressions of IκBα and phosphorylated IκBα proteins in A549 cells following 24 h exposure to PM2.5, as determined by Western blot (**C**) and by densitometric analysis (**D**). (n = 3, *p < 0.05, **p < 0.005).

To confirm the microarray result, we performed real-time PCR. Thus, A549 cells were treated with PM2.5, pollen, and *E*. *coli* K12; of which the latter served as a positive control for Nod1 ligands. Nod1 mRNA expression was increased 2.2-fold following treatment with PM2.5 (2.2 ± 0.48) and about 2-fold following treatment with E. *coli*, (1.96 ± 0.13) compared to untreated cells [Fig. 5A]. In contrast, pollen treatment did not alter the mRNA expression of Nod1 (1.23 ± 0.19) [Fig. 5A].

To assess the adjuvant activity of PMs to antigen inhalation; we investigated the synergistic effect of PM2.5 and pollen on Nod1 activities. By using the Nod1-Flag and NF-κB-dependent GFP reporter bioassay established by Hasegawa *et al*^17^. for screening and characterizing Nod1-stimulatory activities in HEK293-Nod1-GFP cells, we found that PM2.5 + pollen (2.13 ± 0.52) significantly increased the level of Nod1-stimulatory activities in these cells, compared to untreated cells [Fig. 5B]. However, there was no significant difference in Nod-1 activity between PM2.5 (1.88 ± 0.31) and PM2.5 + pollen stimulated cells. In contrast, stimulation with pollen alone did not significantly increase Nod1 activity [Fig. 5B]; indicating that the Nod-1 stimulatory activity was likely to be mostly attributable to PM2.5.

As Nod1 protein is involved in the recognition of bacterial molecules and regulation of innate and acquired immune responses by activating transcription factors including NF-κB, we assessed IκBa and p-IκBa protein levels by Western blot analysis. Both these proteins were induced by treatment with PM2.5, but not by treatment with pollen [Fig. 5C].

## Discussion

Exposure to PM2.5 is closely related to acute and chronic diseases. Herein, we applied a novel approach for investigating the underlying mechanism of cell response to air pollution exposure, by establishing a chronic allergy mouse model and exposing this to the real environment, when the concentrations of PM2.5 are known to be high from September to March in Beijing. Our results showed that exposure of the animals to PM2.5-enriched air pollution exacerbated chronic rhinitis, and resulted in disruption of the nasal mucosal structure and infiltration of inflammatory cells.

To better understand the cell response to ambient air pollution exposure and identify the transcriptomic biomarkers of exposure, genome-wide gene expressions were analyzed by microarray technology. We demonstrated that genes were differentially modulated in response to air pollution in the chronic allergy mouse model; with the gene sets presented in animal in groups CP and AP considered to be specifically regulated by air pollution, compared with control animals in group CC. The genes that were regulated only in group AP among the study groups were considered as biomarkers in chronic allergy.

Exposure to PM2.5 induced the ROS production damaged DNA and triggered inflammatory responses. The function of differentially expressed genes (DEGs) was analyzed to identify gene networks according to the physiological functions and/or diseases in the Ingenuity Pathways Knowledge Database. Our results demonstrated that cell differentiation and maturation, cell-cell signaling, and signal transduction involved in the immune response were the main molecular and cellular functions of genes, which were enriched in the AP group. The findings from the present study also confirm the findings of several other reports, which have suggested that TGF-β signaling was regulated after exposure to PM2.5^18–20^.

The inflammatory potential of PM is partially due to biological components such as bacterial lipopolysaccharides (LPS)^11^, and Camatini and colleagues^21^ have indicated that endotoxin concentration in PM2.5 is higher than in PM10. Exposure to endotoxins and vital biological components from PM2.5 has been reported to be associated with adverse effects on human health^22,23^ and also shown to induce a pro-inflammatory response and necrotic cell death in cultured pulmonary cells and mice lungs^24^. Indeed, while one recent study has demonstrated an association between TREM-1/TREM-1 blockade and the survival of mice with LPS-induced septic shock^25^, another more recent study has suggested that urban PM2.5 bound microbial elements such as LPS may exacerbate allergic inflammation in the murine lung via a TLR2/TLR4/MyD88-signaling pathway^26^. In this regard, the demonstration in the present study that PM2.5 contributed to the modulation of genes such as Nod-like receptor or TLR implicated in pattern recognition receptors for the identification of bacteria, viruses, and signaling in the viruses, suggests that these effects may be possibly be related to endotoxin present in the PM2.5, although this needs to be confirmed in future studies. The innate immune response has been shown to be one of the most significantly overrepresented gene ontology terms for air pollutants, as evaluated by the *Drosophila* model^27^. In this regard, our study showed that PM2.5 induced Nod1 expression and increased IκBα expression and IκBα phosphorylation, thereby suggesting that once the Nods or TLRs signaling pathways are activated, these molecules trigger NF-κB that leads to the activation of transcriptional responses culminating in the expression of a subset of inflammatory genes.

Previously studies have proposed that PM2.5 exposure may exacerbate asthma or allergic rhinitis by acting as an adjuvant that enhances the allergic inflammatory response^28,29^. Furthermore, in addition to chemical substances, the PM2.5-bound microbial substances; such as spores, bacteria, and viruses; are considered to be important in inflammatory and allergic lung diseases^23,30^. The mechanism underlying long-lasting adjuvant effects of fine PM during the induction of allergic inflammation may involve killing of alveolar macrophages, leading to long-lasting release of IL-1α and formation of inducible bronchus-associated lymphoid tissue (iBALT) in the lungs; triggering the local inflammatory response over long periods^7^. We have previously shown that exposure of oak pollen to SO_2_ or NO_2_ resulted in fragility and disruption of the oak pollen, leading to increased release of cytoplasmic pollen granules into the atmosphere. This phenomenon might increase the incidence of allergic airway disease in sensitized individuals by facilitating the bioavailability of airborne pollen allergens^14^. Nod1 has been shown to occur in human nasal epithelium, and its expression in patients with allergic rhinitis was downregulated during pollen season^31^. Some studies have suggested a synergistic effect between virus infection and allergen exposure, and that exposure to microbial products exert a protective effect on allergic asthma^32^. In the present study, we highlighted the dysregulation of the TREM1 (Triggering Receptor Expressed on Myeloid cells1) signaling pathway in synergy with NLRs. This may be of consequence, as TREM1 belongs to the TREM family of receptors of the immunoglobulin superfamily that play critical roles in innate immune responses^33,34^. Moreover, the TREM1 pathway is known to synergize with signals from both major pathways of pattern recognition such as Toll-like receptors (TLRs) and TP-1-leucine-rich repeat receptors (NATCH-LRRs and NLRs)^35,36^ in the production of proinflammatory cytokines. These features conform that environmental pathogens may play a major role in modulating the effect of exposure to air pollution on human health. We also investigated the synergistic effects of PM2.5 and pollen on cellular response, and found that exposure to PM2.5, but not pollen, induced Nod1 expression and increased IκBα expression and IκBα phosphorylation in A549 cells (Fig. 5). Nod1 (CARD4) is an intracellular pattern-recognition molecule, containing nucleotide-oligomerization domain (NOD) and ligand-recognizing leucine-rich repeats, involved in the identification of peptidoglycan. Genetic variation in Nod1 is associated with susceptibility to several inflammatory disorders including allergic diseases and Crohn’s disease^37,38^.

In conclusion, we have developed a chronic allergy mouse model and exposed it to ambient air pollution rich in PM2.5, in order to investigate the effect of PM2.5-induced exacerbation of chronic allergies such as allergic rhinitis. Furthermore, we also explored the mechanism of PM2.5 exposure-induced cell responses, and using whole gene expression profile analysis by DNA microarray revealed the involvement of inflammatory and innate immune response. PM2.5 exposure also activated the innate immunity, induced the expression of pattern recognition receptors with respect to bacteria and viruses, and activated the Nod1 and NF-κB signaling pathways to trigger the pro-inflammatory and immune response. These results provide an insight into the potential effects of ambient air pollution exposure in the chronically allergic individuals, as well as possibly a means to monitor the therapeutic efficacy of drugs employed in treatment of allergic diseases.

## Electronic supplementary material

Supplementary Information
