# Supplementary material for: Changes in gene expression in chronic allergy mouse model exposed to natural environmental PM2.5-rich ambient air pollution
Source: Sci Rep. 2018 Apr 20;8:6326. doi: 10.1038/s41598-018-24831-z (PMC5910422; doi:10.1038/s41598-018-24831-z)
Supplement: Supplementary file 1 — Supplementary Information [file 41598_2018_24831_MOESM1_ESM.pdf]

Changes in gene expression in chronic allergy mouse model exposed to natural environmental PM2.5-rich ambient air pollution

Yuhui Ouyang [1,2#], Zhaojun Xu [3,4#], Erzhong Fan [2], Ying Li [2], Kunio Miyake[5], Xianyan Xu, Luo Zhang [1,2*].

SUPPLYMENTARY FIGURE LEGENDS

Figure S1. Diseases and disorders and molecular and cellular function categories of up-regulated genes in allergic mice following exposure to PM2.5. A. Diseases and disorders, B. Molecular and cellular function.

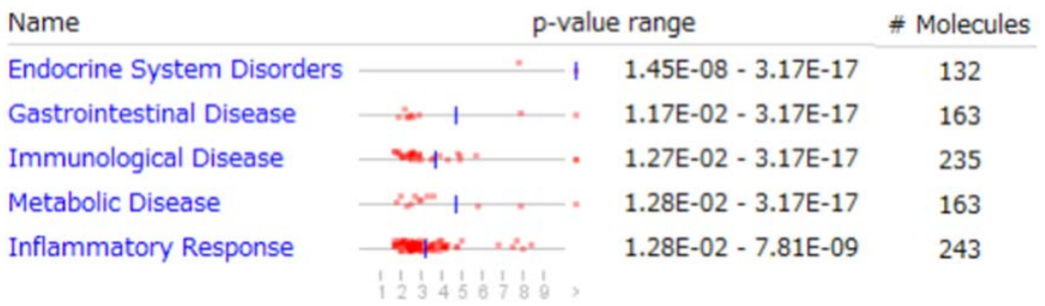

Name	p-value range	# Molecules
Endocrine System Disorders	1.45E-08 - 3.17E-17	132
Gastrointestinal Disease	1.17E-02 - 3.17E-17	163
Immunological Disease	1.27E-02 - 3.17E-17	235
Metabolic Disease	1.28E-02 - 3.17E-17	163
Inflammatory Response	1.28E-02 - 7.81E-09	243

A

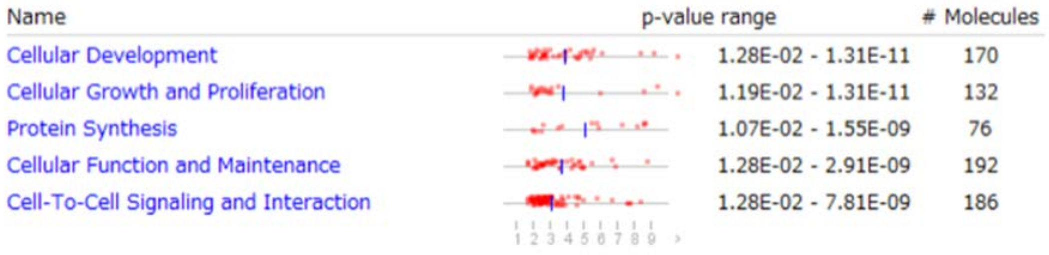

Name	p-value range	# Molecules
Cellular Development	1.28E-02 - 1.31E-11	170
Cellular Growth and Proliferation	1.19E-02 - 1.31E-11	132
Protein Synthesis	1.07E-02 - 1.55E-09	76
Cellular Function and Maintenance	1.28E-02 - 2.91E-09	192
Cell-To-Cell Signaling and Interaction	1.28E-02 - 7.81E-09	186

B

Figure S2. Diseases and disorders and molecular and cellular functional categories of down-regulated genes in allergic mice following exposure to PM2.5. A. Diseases and disorders, B. Molecular and cellular function.

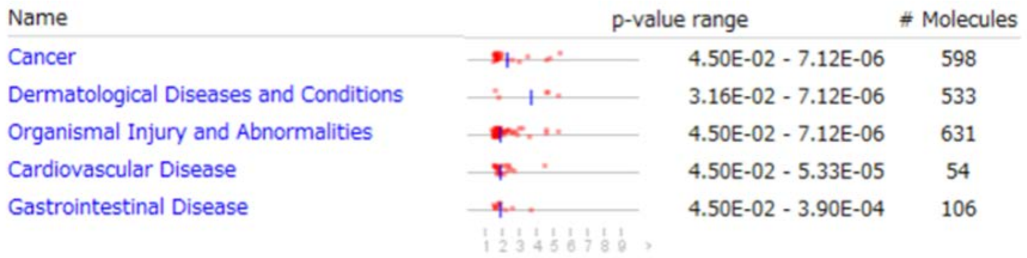

Name	p-value range	# Molecules
Cancer	4.50E-02 - 7.12E-06	598
Dermatological Diseases and Conditions	3.16E-02 - 7.12E-06	533
Organismal Injury and Abnormalities	4.50E-02 - 7.12E-06	631
Cardiovascular Disease	4.50E-02 - 5.33E-05	54
Gastrointestinal Disease	4.50E-02 - 3.90E-04	106

A

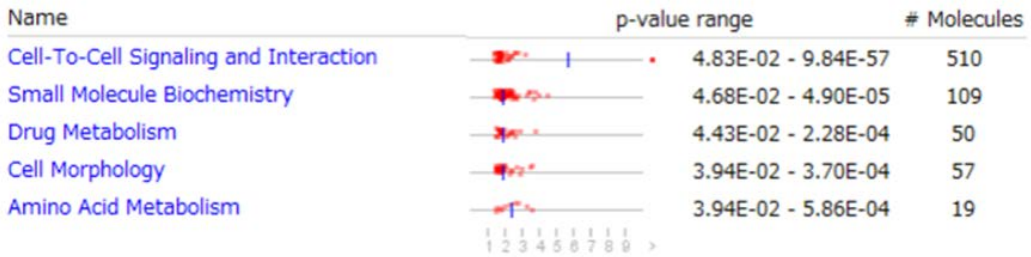

Name	p-value range	# Molecules
Cell-To-Cell Signaling and Interaction	4.83E-02 - 9.84E-57	510
Small Molecule Biochemistry	4.68E-02 - 4.90E-05	109
Drug Metabolism	4.43E-02 - 2.28E-04	50
Cell Morphology	3.94E-02 - 3.70E-04	57
Amino Acid Metabolism	3.94E-02 - 5.86E-04	19

B
